# Supplementary material for: Resolving the full spectrum of human genome variation using Linked-Reads
Source: Genome Res. 2019 Apr;29(4):635–45. doi: 10.1101/gr.234443.118 (PMC6442396; doi:10.1101/gr.234443.118)
Supplement: Supplemental Material [file supp_gr.234443.118_Supplemental_longranger-2.2.zip › longranger-2.2.tar.bz2!/lr2.2-public/lib/stan/cmdstan/stan/doc/api/html/agrad_2rev_2matrix_2mdivide__left__spd_8hpp.html]

Stan: src/stan/agrad/rev/matrix/mdivide\_left\_spd.hpp File Reference


|  |  |
| --- | --- |
|  | Stan  2.5.0  probability, sampling & optimization |


- Main Page
- Related Pages
- Namespaces
- Classes
- Files

- File List
- File Members

All Classes Namespaces Files Functions Variables Typedefs Enumerations Enumerator Friends Macros Pages

- src
- stan
- agrad
- rev
- matrix

Namespaces |
Functions

mdivide\_left\_spd.hpp File Reference

`#include <vector>`  
`#include <stan/math/matrix/Eigen.hpp>`  
`#include <stan/math/matrix/typedefs.hpp>`  
`#include <stan/math/error_handling/matrix/check_multiplicable.hpp>`  
`#include <stan/agrad/rev/var.hpp>`  
`#include <stan/agrad/rev/matrix/typedefs.hpp>`  
`#include <stan/math/error_handling/matrix/check_square.hpp>`

Go to the source code of this file.

|  |  |
| --- | --- |
| Namespaces | |
|  | stan |
|  | Probability, optimization and sampling library. |
|  | |
|  | stan::agrad |
|  | Function gradients via reverse-mode automatic differentiation. |
|  | |

|  |  |
| --- | --- |
| Functions | |
| template<int R1, int C1, int R2, int C2> | |
| Eigen::Matrix< var, R1, C2 > | stan::agrad::mdivide\_left\_spd (const Eigen::Matrix< var, R1, C1 > &A, const Eigen::Matrix< var, R2, C2 > &b) |
|  | |
| template<int R1, int C1, int R2, int C2> | |
| Eigen::Matrix< var, R1, C2 > | stan::agrad::mdivide\_left\_spd (const Eigen::Matrix< var, R1, C1 > &A, const Eigen::Matrix< double, R2, C2 > &b) |
|  | |
| template<int R1, int C1, int R2, int C2> | |
| Eigen::Matrix< var, R1, C2 > | stan::agrad::mdivide\_left\_spd (const Eigen::Matrix< double, R1, C1 > &A, const Eigen::Matrix< var, R2, C2 > &b) |
|  | |

## Variable Documentation

mdivide\_left\_spd\_alloc<R1,C1,R2,C2>\* \_alloc

Definition at line 33 of file mdivide\_left\_spd.hpp.

Eigen::LLT< Eigen::Matrix<double,R1,C1> > \_llt

Definition at line 21 of file mdivide\_left\_spd.hpp.

vari\*\* \_variRefA

Definition at line 30 of file mdivide\_left\_spd.hpp.

vari\*\* \_variRefB

Definition at line 31 of file mdivide\_left\_spd.hpp.

vari\*\* \_variRefC

Definition at line 32 of file mdivide\_left\_spd.hpp.

Eigen::Matrix<double,R2,C2> C\_

Definition at line 22 of file mdivide\_left\_spd.hpp.

int M\_

Definition at line 28 of file mdivide\_left\_spd.hpp.

int N\_

Definition at line 29 of file mdivide\_left\_spd.hpp.


---

[ Stan Home Page ]

*© 2011–2014,
Stan Development Team.*
